# A Novel Biocompatible Ternary Nanoparticle with High Antibacterial Activity: Synthesis, Characterization, and Its Application in Beef Preservation

**DOI:** 10.3390/foods11030438

**Published:** 2022-02-02

**Authors:** Lin Lin, Chencheng Luo, Changzhu Li, Xiaochen Chen, Haiying Cui

**Affiliations:** 1 School of Food and Biological Engineering, Jiangsu University, Zhenjiang 212013, China; linl@ujs.edu.cn (L.L.); kjld2008@126.com (C.L.); 1000005533@ujs.edu.cn (X.C.); 2 State Key Laboratory of Utilization of Woody Oil Resource, Hunan Academy of Forestry, Changsha 410007, China; lichangzhu2013@aliyun.com

**Keywords:** ternary nanoparticle, encapsulation efficiency, stability, antimicrobial activity, antioxidant activity

## Abstract

Edible nanoparticles containing antibacterial agents are one of the effective strategies to control foodborne diseases. Herein, novel ternary nanoparticles (TNP) were prepared from rosemary essential oil (REO), nisin and Lycium barbarum polysaccharides (LBP) through hydrophobic and electrostatic interaction. The average particle size of TNP was 211.5 nm, and its encapsulation efficiency reached 86.6%. After the addition of LBP, the physical stability, thermal stability and storage stability of TNP were significantly improved. In vitro, compared with the control group, the population of *S. aureus* and *E. coli* O157:H7 in the TNP-treated group was reduced by 2.386 log CFU/mL and 1.966 log CFU/mL, respectively, on the fifth day. The free radical scavenging rate of TNP was 63.15%. The application of TNP on beef presented favorable preservation effects without affecting its color and texture. Therefore, the synthesis strategy of TNP has important reference significance for the research and development of new food antibacterial agents.

## 1. Introduction

Foodborne diseases caused by foodborne pathogenic microorganism contamination have high morbidity and mortality worldwide [1,2]. According to WHO statistics, about 600 million cases of foodborne diseases occur every year, and more than 420,000 people lose their lives as a result [3]. Foodborne pathogenic microorganisms mainly include orally transmitted bacteria, viruses, and parasites. Among them, foodborne pathogens, such as *Staphylococcus aureus*, *Salmonella*, *Listeria monocytogenes* and *Escherichia coli* played the most critical role in food safety problems [4]. In recent years, in order to overcome the shortcomings of traditional food antibacterial strategies, the development of new packaging and coating materials containing natural antibacterial agents based on nanotechnology has become one of the new strategies to reduce foodborne diseases [5]. Nanocarrier technologies including nanoparticles, nanoemulsions, nanohydrogels, nanoliposomes and nanofibers have been widely used in the field of food safety [6]. Nikolic et al. synthesized metal oxide nanoparticles as intelligent food packaging and found that metal oxide packaging exhibited better antibacterial potential [7]. Chitosan nanoemulsion can be used to preserve the quality of muscle foods [8]. Fucinos et al. studied smart nanohydrogels for controlled release of food preservatives [9]. Our research group has extensively studied the effect of nanoliposomes and nanofibers on prolonging the action time of essential oils and the preservation effect of nanoliposomes and nanofibers in food application [10,11,12,13].

As a new type of food additive, functional nanoparticles with food-grade biological macromolecules such as protein and polysaccharides as the main components have been extensively studied because of their large specific surface area, good biocompatibility, simple preparation process, and flexible structure [14,15]. In order to further improve the stability of single-component nanoparticles based on protein or polysaccharides and increase their encapsulation efficiency of active ingredients, the development of multi-component functional nanoparticles has attracted more and more attention [16]. Li et al. synthesized lutein/zein/soybean polysaccharide nanoparticles and found that the encapsulation efficiency was more than 80%. Meanwhile, the nanoparticles showed excellent pH stability and salt ion tolerance [17]. Wang et al. prepared curcumin/sodium caseinate/soybean polysaccharide nanoparticles and found that sodium caseinate had a stronger binding effect with curcumin, while soybean polysaccharide improved the environmental stability of nanoparticles [18]. Although these functional nanoparticles composed of a biologically active ingredient and two carrier molecules significantly improved the stability of nanoparticles, their activity duration is relatively short, and there is room for further improvement of antibacterial activity.

Herein, a novel ternary nanoparticle (TNP) containing two active ingredients and a carrier material were prepared from rosemary essential oil (REO), nisin and Lycium barbarum polysaccharides (LBP). Studies on this type of nanoparticle are still relatively few. As the main active ingredient, REO has good antibacterial and antioxidant effect [19,20,21], and can be considered as a suitable green alternative synthetic antibacterial agent to inhibit pathogens in food. Nisin is adopted not only as another active ingredient to achieve antibacterial and antioxidant effect [22,23,24], but also a peptide-based material of nanoparticles, encapsulating REO through hydrophobic interaction [25]. However, nisin easily interacts with food ingredients and is easily degraded by enzymes [26,27,28], which can be solved by combining nisin with biopolymers or by synthesizing nisin-loaded nanoparticles [29,30,31,32]. As a natural Chinese herbal medicine polysaccharide, LBP has a variety of biological activities, good biocompatibility, good selectivity, and low cytotoxicity [33,34,35]. In addition, Liu et al. (2021) found that LBP has a highly branched chain structure and is rich in hydroxyl groups, which can be used as a potential stabilizer for nanoparticles [36]. Therefore, LBP has the potential to be a stabilizer for REO/nisin nanoparticles (RNNP), which were combined with nisin by electrostatic interaction. In summary, in view of the instability of protein nanoparticles, we use a polysaccharide to modify the protein to improve the stability of the nanoparticles. On this basis, we prepared TNP with high encapsulation efficiency and high stability. Compared with most nanoparticles, the TNP prepared in this paper has dual active components in order to improve its antibacterial and antioxidant effect. We also demonstrated the improvement of the stability of TNP and its practical value through thermal stability, storage stability and application in beef.

## 2. Materials and Methods

### 2.1. Materials and Bacterial Culture

*Staphylococcus aureus* (*S. aureus*) ATCC 25,923 and *Escherichia coli EHEC* O157:H7 (*E. coli* O157:H7) CICC 21,530 were provided by the Beina Institute of Biotechnology (Beijing, China). The nutrient agar (NA) and nutrient broth (NB) were chosen as mediums and were cultivated at 37 °C for 1 day. Rosemary essential oil (REO) was bought from JE International (Natural CBD, Shanghai, China). Nisin was provided by Zhejiang Silver Elephant Bioengineering Co., Ltd. (Taizhou, China). Lycium barbarum polysaccharides (LBP) were purchased from Jiangzhang Industrial Park (Baoji, China). Beef was purchased from Kaiyuan Supermarket (Zhenjiang, China).

### 2.2. Synthesis of TNP

LBP (3 mg/mL) was dissolved in distilled water and stirred for 120 min. Next, nisin (3 mg/mL, pH = 4) was slowly dropped into the LBP solution and stirred for 1 h. The mixed solution was centrifuged (3500× *g*, 10 min). The REO (2 Minimum Inhibitory Concentration, 2MIC) was dropped into the solution and stirred for 30 min. Finally, it was ultrasonicated for 10 min (600 W, duration 1 s, intervals 1 s). Different nanoparticles were prepared by the above method: nanoparticles-I (LBP:nisin = 0:1, v:v), nanoparticles-II (LBP:nisin = 1:3, v:v), nanoparticle-III (LBP:nisin = 1:1, v:v), nanoparticles-VI (LBP:nisin = 3:1, v:v), nanoparticles-V (LBP:nisin = 5:1, v:v).

### 2.3. Characterization of TNP

#### 2.3.1. Particle Size, PDI and Encapsulation Efficiency of Nanoparticles

The particle size and polymer dispersity index (PDI) of different nanoparticles were measured by a laser particle size analyzer (Nano ZS90, Malvern Instruments, Worcester, UK). The standard curve of REO was obtained through GC-MS (6890 GC/5973 NMSD, Agilent, Palo Alto, CA, American) [37]. The initial temperature was 60 °C for 1 min, then the temperature was raised to 120 °C at 4 °C/min and raised to 150 °C at 10 °C/min for 5 min. Finally, the temperature was raised to 250 °C at the same rate and kept for 3 min. The essential oil concentrations were 1, 2, 4, 6 and 8 μL/mL, and the injection volume was 1 μL. The concentration of REO embedded in different nanoparticles was determined by GC-MS. The following Formula (1) obtained the encapsulation efficiency (EE%) of the nanoparticles.

EE% = A/T × 100%(1)

where A is the amount of essential oil contained and T is the total essential oil content [38].

#### 2.3.2. The Morphology of Nanoparticles

Scanning electron microscopy (SEM) (COXEM EM-30 Plus, Daejeon, Korea) and an atomic force microscope (AFM) (Multimode 8 of Bruker, Mass) were used to observe the morphology of nanoparticles. The lyophilized nanoparticles (nanoparticles-I (LBP:nisin = 0:1, v:v) and nanoparticle-III (LBP:nisin = 1:1, v:v)) were rehydrated. They were then evenly dropped on the silicon wafer and dried with liquid nitrogen. SEM observed the morphology of the gold sprayed samples. The sample pre-treatment of AFM was the same as that of SEM. The rehydrated nanoparticles were dropped onto a mica sheet and observed with AFM.

#### 2.3.3. UV-Vis Absorption Spectrum

UV-vis absorption spectrum was used to analyze the hydrophobicity of substances. The UV spectra was scanned by spectrophotometer (UV-1801, Rayleigh, Beijing, China). The scanning range was 190–900 nm, and the scanning interval was 1 nm. 

#### 2.3.4. Fluorescence Spectroscopy Measurement

A fluorescence spectrophotometer (F-4500, Hitachi, Japan) was used to scan the fluorescence spectrum. The excitation wavelength was 280 nm, the wavelength range was 300–500 nm, the scanning speed was 1000 nm/min, and the excitation bandwidth was 10 nm.

#### 2.3.5. Fourier Transform Infrared Spectroscopy (FTIR) Analysis of Nanoparticles

In order to analyze the interaction between nisin and LBP, and whether REO was encapsulated by nanoparticles, we carried out a Fourier transform infrared spectroscopy (FTIR). The scanning range of the infrared spectrum was 800–4000 cm^−1^, and the scanning times were 32 [39]. 

#### 2.3.6. Quartz Crystal Microbalance (QCM)

To verify the encapsulation effect of nisin nanoparticles (NNP) on REO and the modification effect of LBP on RNNP, the minor mass differences among NNP, RNNP, and TNP were analyzed by quartz crystal microbalance (QCM) (Q-Sense E4, Baiolin, Swedish). The solvent was deionized water with a flow rate of 100 μL/mL. The small mass differences between the three nanoparticles were measured in terms of frequency (ΔF) and energy loss(ΔD) [40]. 

#### 2.3.7. X-ray Diffraction (XRD)

The X-ray diffraction (XRD) was carried out at 40 kV and 40 mA by X-ray diffractometer (D8 ADVANCE, Brook, Germany) to analyze the crystallinity of nanoparticles. The scanning range of the diffraction pattern was 4–90° (2θ), and the scanning rate was 5.0°/min [41].

### 2.4. Stability of Nanoparticles

The thermogravimetric analysis (TGA) and differential scanning calorimetry (DSC) were analyzed by a thermal analyzer (STA449F3, NETZSCH, Selb, Germany). The sample (5 mg) was weighed and heated from room temperature to 800 °C. The temperature rose at 10 °C/min, and the weight loss of each sample was recorded [42].

The particle size and PDI of nanoparticles were determined under different pH (2–7) and ionic strength (NaCl concentration: 0–100 mmol/L) to observe the physical stability of nanoparticles. TNP were stored at 4 °C. The particle size and PDI were measured at 0, 5, 10, 15, 20, 25 and 30 days to analyze the storage stability of nanoparticles.

### 2.5. Antibacterial Effect of Nanoparticles

The antibacterial effect of nanoparticles was evaluated by the inhibition zone, transmission electron microscope (TEM), and time-kill curve. First, we took 100 μL bacterial solution (bacteria concentration 10^6^–10^7^) and coated the plate, then a filter paper with a diameter of 10 mm was stuck in the middle of the plate. Nanoparticles (10 μL) were dropped on the filter paper, and put it into the incubator. The size of the inhibition zone was measured. 

The structure of bacteria was measured by TEM. The bacteria (bacteria concentration 10^4^–10^5^) were treated with different nanoparticles (RNNP and TNP) for 4 h, the concentration of nanoparticles was 40%, and the bacteria not treated with nanoparticles were used as control. Next, each sample was centrifuged at 8000 rpm for 5 min. Then the copper mesh was immersed in the washed bacterial suspension, left for 3 min, and dried. The copper mesh with the bacteria was then stained in a 3% (w/w) phosphotungstic acid solution for 15 min and dried. Finally, the product was observed by TEM (JSM-7001F, Japan).

The bacteria (bacteria concentration 10^4^–10^5^) were treated with different nanoparticles (RNNP and TNP), the concentration of nanoparticles was 40%, and the bacteria not treated with nanoparticles were used as control. The plate colony counting method was used to record the daily changes in the number of bacteria to reflect the bactericidal effect of the nanoparticles within five days.

### 2.6. Antioxidant Effect of Nanoparticles

#### 2.6.1. DPPH Scavenging Activity

Different samples (1 mL) were mixed with 3 mL DPPH (0.1 mmol/L) and then were placed in the dark for 30 min and the absorbance at 517 nm were measured by spectrophotometer. The antioxidant activity can be obtained by the following Formula (2).

Antioxidant activity = [1 − (A_2_ − A_1_)/A_0_] × 100% (2)

A_0_ was the absorbance of blank, A_2_ was the absorbance of the sample reacted with DPPH, and A_1_ was the absorbance of the sample reacted with ethanol [43].

#### 2.6.2. Antioxidant Effect on Beef

Mix beef (3 g) from different treatment groups with trichloroacetic acid solution (TCA, 15 mL) were beaten with a sterile homogenizer for 2 min, and filtered. The filtrate was mixed with thiobarbituric acid solution (TBA, 5 mL) and then heated in a water bath (100 °C) for 30 min. The mixture was centrifuged after cooling to room temperature. The absorbance of the supernatant was measured at 532 nm. The TBA of the sample was calculated according to the following Formula (3) [44].

TBA (mg/kg) = A/m × 9.48(3)

Different beef homogenate samples were mixed with 30 mL of sterile water, and were incubated with shaking at 37 °C for 30 min. After filtering and centrifuging the mixture, 10 mL of the supernatant was taken and mixed with an equal volume of MgO solution (1%, w:v). After distilling for 5 min, the boric acid solution (2%, w:v) containing 5–6 drops of methyl red-methylene blue mixed indicator was used to absorb the volatile nitrogenous substances in the distillate. A hydrochloric acid solution (0.01 mol/L) was used to titrate the above-mentioned boric acid solution containing volatile nitrogen substances. The volume of hydrochloric acid solution consumed was recorded. An equal volume of deionized water replaced the sample supernatant as a blank test, and the following Formula (4) was used to calculate the TVB-N value in different samples:

TVB-N (mg/100 g) = {[(V_1_ − V_2_) × C × 14]/(m × 5/100)} × 100 (4)

V_1_ and V_2_ were the volume of the hydrochloric acid solution consumed by the sample and blank (mL), individually, C was the concentration of the hydrochloric acid solution (mol/L), m was the mass of the sample (g), and the conversion factor was 14 [44].

### 2.7. The Application of Nanoparticles against S. aureus and E. coli O157:H7 on Beef

The beef was cut into equal sized pieces (mass about 3 g). It was soaked in nanoparticles for 10 min at room temperature, then an appropriate number of bacteria (10^4^–10^5^) were added to the beef. The treated beef was placed at 4 and 25 °C for 5 days. The plate colony counting method was used to record the daily changes in colonies.

### 2.8. Characterization of Coated Beef

The color, pH and texture of beef in different treatment groups were also recorded. The color was performed by a Colorimeter (3 nh, Shenzhen sanenshi Technology Co., Ltd., Shenzhen, China). The pH was measured using a pH meter according to Chinese standard GB 5009.237-2016. Surface texture measurement of the beef was performed using a texture analyzer (ST-Z16, Shandong Shengtai Instrument Co., Ltd., China). The specific parameters were as follows: the test speed was 5.00 mm/s, the strain was 50%, and the trigger force was 5.0 g.

### 2.9. Statistical Analysis

Three parallel groups were set in each group. The results were analyzed by SPSS (version 26.0; IBM Corp., Armonk, NY, USA), which was expressed as mean ± standard deviation (SD).

## 3. Results and Discussion

### 3.1. Characterization of Nanoparticles

#### 3.1.1. Particle Size, PDI and Encapsulation Efficiency of Nanoparticles

The results of particle size, PDI and morphology of nanoparticles showed that TNP were successfully prepared. Table 1 showed the particle size, PDI, and encapsulation efficiency of nanoparticles with different ratios, which are metrics for measuring nanoparticles [45]. When the particle size of nanoparticles is too tiny, nanoparticles are easy to agglomerate. When the particle size of nanoparticles is too large, the drug loading effect of the nanoparticle is not good. Nanoparticle I was RNNP with a particle size of 167.8 ± 4.8 nm, PDI of 0.242 ± 0.008, and encapsulation efficiency of 53.7% ± 0.6. Nanoparticles II to V were TNP, and the particle size became larger after the modification of binary nanoparticles by polysaccharides, ranging from 211.5 ± 8.1 nm to 386.5 ± 2.3 nm, indicating that polysaccharides successfully modified the RNNP. When nisin was more abundant, the particle size was 386.5 ± 2.3 nm, PDI was 0.302 ± 0.022, and the encapsulation efficiency was lowest at 84.4% ± 0.3. These results showed that the formed TNP were uneven in distribution and large in size. This distribution was due to the fact that there were not enough LBP to modify nisin. The clumping of nisin occurred, and the hydrophobic effect between nisin and REO was weak. When the ratio of nisin to LBP was 1:1, the particle size was the smallest, the encapsulation efficiency was the highest, and the PDI was less than 0.3, so nisin: LBP = 1:1 was the optimal ratio. At this time, LBP fully modified nisin to expose more hydrophobic bonds, which enhanced the hydrophobic effect between nisin and REO. When LBP was in excess of nisin, the particle size began to increase. This increase was due to the accumulation of excess LBP on the surface of nisin. PDI indicated the degree of dispersion of nanoparticles; except for nanoparticle II, the PDI of the rest of the nanoparticles were all less than 0.3, indicating that the nanoparticles had good dispersion. Encapsulation efficiency is one of the most critical indicators of nanoparticles. Compared with RNNP, the different ratios of the prepared TNP all showed improved encapsulation efficiency, ranging from 84.4% ± 0.3 to 86.6% ± 0.2. These results indicated that nisin nanoparticles modified by LBP could improve their encapsulation efficiency.

#### 3.1.2. Morphology Analysis of Nanoparticles

**Figure 1:**
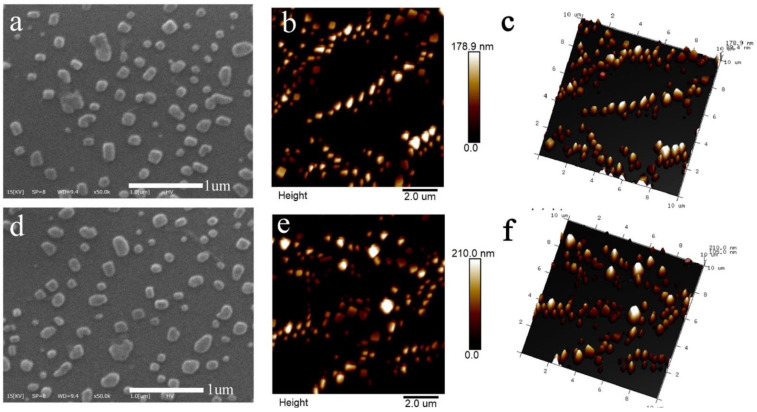
The morphology of nanoparticles including SEM-micrographs and surface, 3-Dimensional AFM images of RNNP (**a**–**c**) and TNP (**d**–**f**).

The SEM-micrographs and AFM images of RNNP and RNNP modified by polysaccharides were presented in Figure 1. The two kinds of nanoparticles were uniformly spherical. After the modification of nanoparticles with polysaccharides, the particle size of RNNP became larger.

#### 3.1.3. UV-Vis Spectral Analysis

**Figure 2:**
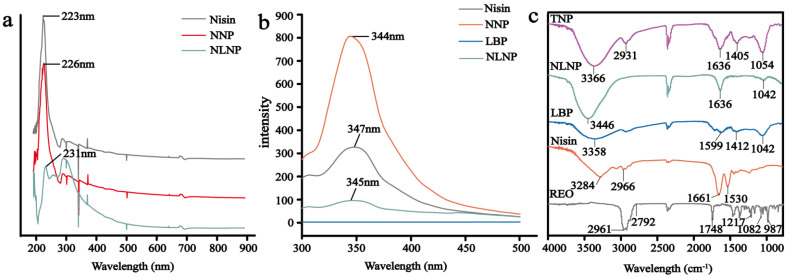
The spectral analysis of nanoparticles including UV-vis absorption spectrum(**a**) and Fluorescence spectroscopy(**b**) of Nisin, NNP and NLNP; FTIR spectrum(**c**) of REO, Nisin, LBP, NLNP and TNP.

The increase of the encapsulation efficiency of TNP may be due to the more hydrophobic bonds exposed by the interaction between protein and polysaccharide. The results of UV and fluorescence spectra were consistent with our conjecture. The increase of encapsulation efficiency was due to more water dispersing bonds exposed by the interaction of protein and polysaccharide. In addition, we found other results. As shown in Figure 2a, the maximum absorption peak of nisin appeared at 223 nm, consistent with previous studies [46]. The absorption peaks of NNP and nisin/LBP nanoparticles (NLNP) were red-shifted to 226 nm and 231 nm, respectively, which may be due to the enhanced hydrophobicity of nanoparticles [47]. In addition, the NLNP red-shifted to a greater extent than NNP, which indicated that NLNP exposed more hydrophobic bonds. These results further proved that nisin modified by polysaccharides could improve the encapsulation efficiency of nanoparticles.

#### 3.1.4. Fluorescence Spectroscopy Analysis

As was shown in Figure 2b, the fluorescence peak of nisin appeared at 347 nm, which was due to the existence of tryptophan residues. The result was consistent with Narahari et al. [48]. The fluorescence intensity of LBP was 0, indicating that LBP had no fluorescence. Compared to free nisin, the fluorescence intensity of NNP was enhanced from 326.5 to 806.5, which might be related to the enhanced conjugation degree when nanoparticles were formed. The fluorescent intensity of NLNP was weakened, which was attributed to the polysaccharides being barely band fluorescent. These results illustrated that nisin was encapsulated in the polysaccharides. The wavelengths corresponding to the fluorescence maxima of NNP and NLNP were blue-shifted from 347 nm to 344 nm and 345 nm, respectively. This can be the result of the exposure of tryptophan residues to a more hydrophobic environment [49]. In addition, more hydrophobic bonds were exposed when nisin formed nanoparticles or nisin and LBP were combined to form complexes. This result was consistent with Li et al. [50]. The fluorescence spectra results illustrated that the binding of LBP to nisin had a certain effect on the microenvironment of tryptophan and even nisin. Furthermore, when nisin was made into nanoparticles or modified with polysaccharides, the hydrophobicity of NNP and NLNP could be increased to improve the encapsulation efficiency of essential oil. The fluorescence results were consistent with the results of the UV spectrum. In addition, the microenvironment with the enhanced hydrophobicity of protein can improve the stability of protein [51]. Therefore, nisin modified by LBP not only improved the encapsulation efficiency of nanoparticles, but also improved its stability.

#### 3.1.5. Infrared Spectroscopic Analysis of Nanoparticles

The main reasons for the formation of TNP are hydrophobic interaction and electrostatic interaction. The results of FTIR presented the interaction between the materials during the formation of nanoparticles. Figure 2c presented the FTIR spectrum of REO, nisin, LBP, NLNP and TNP. As shown in the FTIR spectrum of REO, from 2795 cm^−1^ to 2961 cm^−1^ there were C-H stretching bands. 1748 cm^−1^ was the peak of the keto of camphor. The peak at 987 cm^−1^ and 1217 cm^−1^ was due to the ether function of an epoxy ring of 1, 8-cineole. The peak at 1082 cm^−1^ was related to the asymmetric stretching of the C-O bond [52,53]. The FTIR spectrum of nisin showed that the peak at 3284 cm^−1^ was due to the axial tensile vibration of O-H and N-H. The peak at 2966 cm^−1^ was attributed to the tensile vibration of C-H. The peak at 1661 cm^−1^ was the absorption peak of the amide band, and the peak at 1530 cm^−1^ was related to the bending of the primary amine [54]. The peaks of LBP appeared at 1045 cm^−1^ (C-O), 1599 cm^−1^ (COO- stretching) and 3358 cm^−1^ (O-H stretching vibration), which were also the common characteristic peaks of polysaccharides [55]. The N-H stretching peak of nisin in NLNP was at 3449 cm^−1^, and the N-H stretching peak of nisin was at 3284 cm^−1^. This was due to the new intramolecular hydrogen bond between the N-H group of nisin and the O-H stretching of LBP [56]. In addition, the COO- changed from 1599 cm^−1^ to 1636 cm^−1^, and the primary amine peak of nisin changed from 1530 cm^−1^ to 1636 cm^−1^. It indicated that there was electrostatic interaction between nisin and LBP, which was one of the crucial reasons for the formation of nanoparticles [54]. Compared with the infrared spectra of NLNP, TNP had a characteristic peak at 2932 cm^−1^, which was the C-H stretching band of REO, and the absorption peak at 1054 cm^−1^ was enhanced. These results showed that NLNP successfully encapsulated REO.

#### 3.1.6. QCM

**Figure 3:**
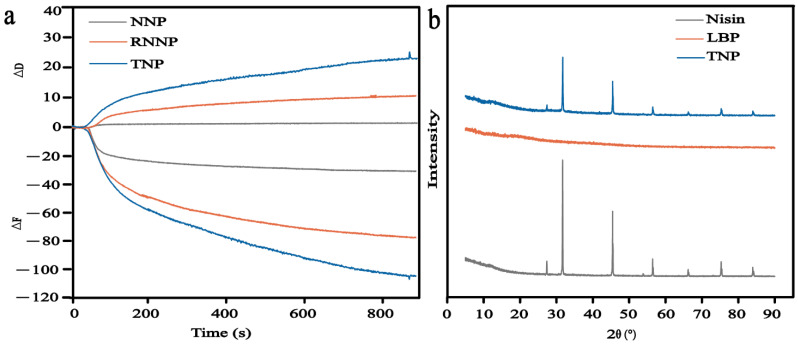
QCM of NNP, RNNP and TNP (**a**); XRD of Nisin, LBP and TNP(**b**).

The results of QCM further showed that REO was successfully embedded in nanoparticles [57]. As was shown in Figure 3a, the changes of frequency (ΔF) and energy loss (ΔD) showed that the three types of nanoparticles had good adsorption with the gold carrier. The energy loss of the three types of nanoparticles changed little, and the frequency changed significantly. Compared with NNP, the ΔF and ΔD of RNNP became larger. These results showed that REO was successfully embedded in NNP, resulting in the increase of its mass. Compared with RNNP, the mass of TNP increased. These results showed that LBP modified RNNP.

#### 3.1.7. XRD

Figure 3b was XRD of nisin, LBP, and TNP, which can analyze the crystallinity of the substance. The diffraction peaks of nisin were found at 32°, 46° and 56°. These results showed that nisin was crystalline, which was consistent with the results of Chang et al. [47]. The XRD pattern of LBP was a straight line, which proved that LBP was an amorphous structure. The XRD pattern of TNP was the same as that of nisin, indicating that TNP did not damage the crystal structure of nisin. However, the intensity of three diffraction peaks (32°, 46° and 56°) of TNP all showed a decreasing trend. On the one hand, the original absorption peak of nisin was weakened due to the connection of nisin and REO after encapsulation. The inclusion of REO with nisin formed a new phase. On the other hand, nisin was encapsulated in LBP, and there was electrostatic interaction between them.

### 3.2. The Stability of Nanoparticles

**Figure 4:**
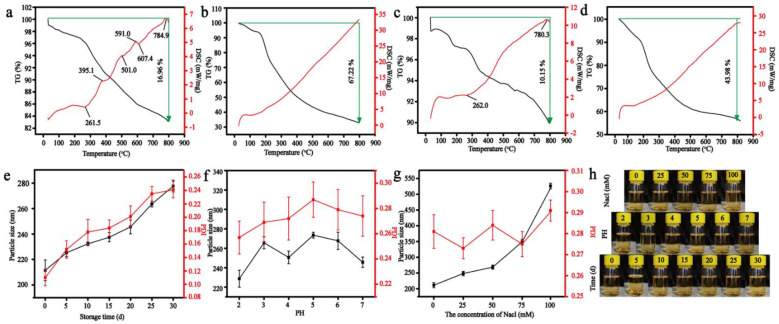
The stability of nanoparticles including the thermal stability of nanoparticles including TGA and DSC of Nisin (**a**), LBP (**b**), RNNP (**c**) and TNP (**d**) and the storage stability (**e**), physical stability (**f**,**g**) and picture (**h**) of TNP.

The thermal stability of nanoparticles was evaluated by TGA and DSC. The results were presented in Figure 4. As can be seen from Figure 4a, nisin had strong thermal stability. When it was heated to 800 °C, the weight loss was 16.96%. The results were consistent with those of Zhang et al. [58]. At 296.3 °C, the weight loss rate reached the maximum, and the weight loss was about 5.13%. Figure 4b was the TGA diagram of LBP, and its weight loss was 67.22%. At 197.4 °C, the weight loss rate was the highest, and the weight loss was 16.63%. Figure 4c,d were the thermogravimetry diagrams of RNNP and TNP, respectively. Compared with the pure materials, the weight loss was reduced by 10.15% and 43.98%, respectively. These results proved that when nisin and LBP were prepared into nanoparticles, their thermal stability was improved. In addition, the prepared nanoparticles had high thermal stability. The weight loss of LBP was 50.26% more than that of nisin. The weight loss of TNP was 33.83% more than that of RNNP, which was due to the fact that LBP was the outermost layer of TNP, and the weight loss of the polysaccharide was more significant at 800 °C. However, this value was lower than the increase in the weight loss of pure substance. It proved that the thermal stability of the nanoparticles could be improved by modifying RNNP with LBP. Compared with the DSC of nisin, the endothermic and exothermic behaviors of RNNP disappeared in the range of 300–600 °C. The DSC of TNP was similar to that of LBP, and there was no endothermic and exothermic peak of nisin. These results indicated that nisin was encapsulated in LBP.

Storage stability and physical stability were also used to evaluate the stability of TNP. The storage stability of TNP was shown in Figure 4a. With the increase of storage days, the particle size and PDI of TNP showed an increasing trend. On 0 d, the particle size of TNP was 211.5 ± 8.1 nm, and the PDI was 0.110 ± 0.012. The particle size of TNP was 277.7 ± 4.6 nm, and the PDI was 0. 241 ± 0.012 after being stored at 4 °C for 30 days. The particle size increased by 66 nm. The change of particle size was small, and the PDI was less than 0.3. These results indicated that the prepared TNP were more stable. Figure 4b showed the particle size and PDI of TNP at different pH. The maximum particle size was 273.5 ± 2.9 nm when the pH was 5. The minimum particle size was 228.7 ± 8.7 nm when the pH was 2. The particle size of TNP at different pH was less than 300 nm, and the PDI was less than 0.3. These results proved that TNP were not sensitive to pH. Figure 4c presented the stability of TNP in different salt ionic strengths. With the increase of salt ionic strength, the particle size of TNP also increased. When the concentration of sodium chloride was 75 mmol/L, the particle size of TNP was 349.2 ± 7.3 nm. When the concentration of sodium chloride was 100 mmol/L, the particle size of TNP was 526.1 ± 8.7 nm. These results showed that when the concentration of sodium chloride was less than 75 mmol/L, TNP were more stable.

### 3.3. Antimicrobial Effect of Nanoparticles

**Figure 5:**
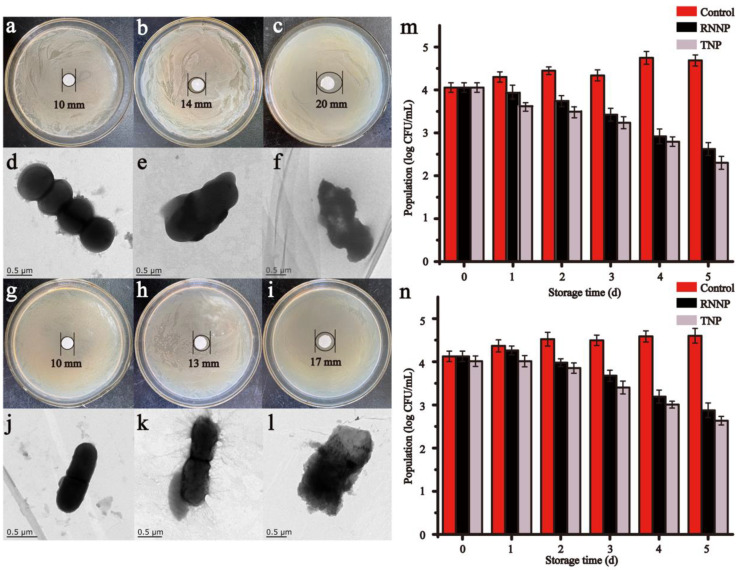
The antibacterial effect of nanoparticles including the inhibition zones, TEM and time-kill curve of control, RNNP and TNP on *S. aureus* (**a**–**f**,**m**) and *E. coli* O157:H7 (**g**–**l**,**n**).

The antibacterial effects of two kinds of nanoparticles on *S. aureus* and *E. coli* O157:H7 and the TEM were presented in Figure 5. Both types of nanoparticles showed good antimicrobial activity. The inhibition zones of RNNP on *S. aureus* and *E. coli* O157:H7 were 14 mm (Figure 5b) and 13 mm (Figure 5h), respectively. When the nanoparticles were modified with polysaccharides, the inhibition zones increased to 20 mm (Figure 5c) and 17 mm (Figure 5i). These results showed that the antibacterial activity of TNP was enhanced, and the result was consistent with the increase in the encapsulation rate.

TEM images of bacteria showed similar results. Compared with RNNP, TNP caused more damage to bacteria. Figure 5d,j were TEM images of bacteria not treated with antimicrobial agents. The surface of the bacteria was smooth. Figure 5e,k were TEM images of bacteria treated with RNNP. The surface of the bacteria was slightly rough. Figure 5f,l were TEM images of bacteria treated with TNP, and the bacteria were destroyed entirely, and the contents were delivered.

The results of the time-kill curve further proved that the antibacterial effect of TNP was better than that of RNNP. The initial number of *S. aureus* and *E. coli* O157:H7 was 4.054 log CFU/mL and 4.125 log CFU/mL, respectively. The number of *S. aureus* and *E. coli* O157:H7 were reduced by 2.064 log CFU/mL and 1.727 log CFU/mL, respectively by RNNP, and TNP reduced the number of *S. aureus* and *E. coli* O157:H7 by 2.386 log CFU/mL and 1.966 log CFU/mL, respectively within five days (Figure 5m,n).

### 3.4. Antioxidant Effect of Nanoparticles

#### 3.4.1. DPPH Scavenging Ability

**Figure 6:**
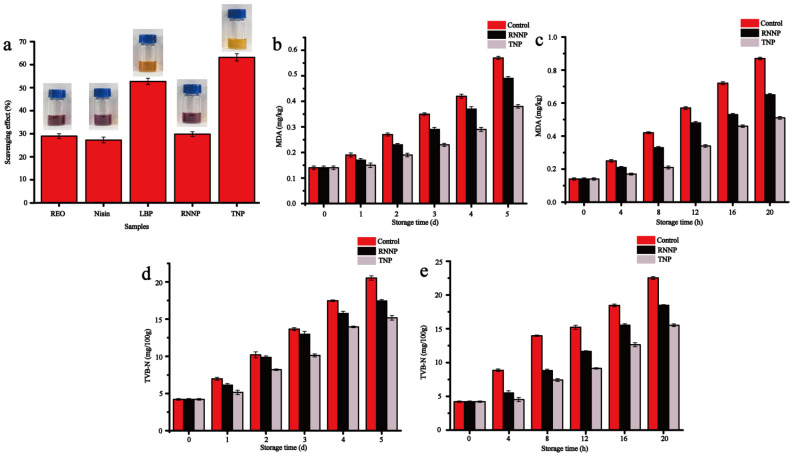
The antioxidation effect of nanoparticles including DPPH free radical scavenging capacity (**a**), TBA (**b**, **c**) and TVB-N (**d**, **e**) at 4 °C and 25 °C.

The DPPH scavenging ability of nanoparticles and their antioxidant effects on beef were presented in Figure 6. The highest DPPH scavenging ability among the five samples was observed in TNP, which was 33.3% higher than that of RNNP (Figure 6a). On the one hand, it was owed to the strong DPPH scavenging ability of LBP that reached 52.71%. On the other hand, it was due to the higher encapsulation rate of TNP.

#### 3.4.2. Antioxidant Effect on Beef

TBA can be used to evaluate the degree of fat oxidation in beef during storage. The MDA values of beef increased throughout the storage period, and the MDA values of the samples treated with nanoparticles were significantly reduced compared with the control samples. The MDA values of the control samples increased from 0.14 mg/kg to 0.57 mg/kg at 4 °C. The MDA values of beef treated with TNP were the lowest. These results were consistent with the strong DPPH scavenging ability of TNP. Compared with the control samples, the MDA values of the samples treated with RNNP and TNP decreased by 14.04% and 33.33% at 4 °C, respectively (Figure 6b). The MDA values of the samples treated with RNNP and TNP decreased by 25.29% and 41.38% at 25 °C, respectively (Figure 6c).

TVB-N is a key factor for evaluating beef freshness by the degree of protein oxidation [59]. GB 2707-2016 stipulates that the TVB-N values of fresh meat are less than 15 mg/100g. The TVB-N values of beef in different treatment groups were shown in Figure 6d,e. With the increase of storage time, the TVB-N values of all samples showed an upward trend. However, the values of the samples treated with nanoparticles were significantly lower than that of the control samples, and the antioxidant effect of TNP was better than that of RNNP. When the beef was stored at 4 °C for 5 days, the TVB-N value of the control samples reached 17.47 mg/100 g on the fourth day, indicating that the beef was not fresh. The beef treated with TNP remained fresh on the fifth day (Figure 6d). When the beef was stored at 25 °C, the TVB-N values of control samples exceeded 15 mg/100 g at the 12th hour, the beef treated with RNNP reached this value at the 16th hour, and the beef treated with TNP was still fresh at the 20th hour (Figure 6e).

These results indicated that LBP enhanced the antioxidant effect of RNNP, and TNP played an important role in delaying lipid oxidation and protein oxidation.

### 3.5. The Application of Nanoparticles against S. aureus and E. coli O157:H7 on Beef

**Figure 7:**
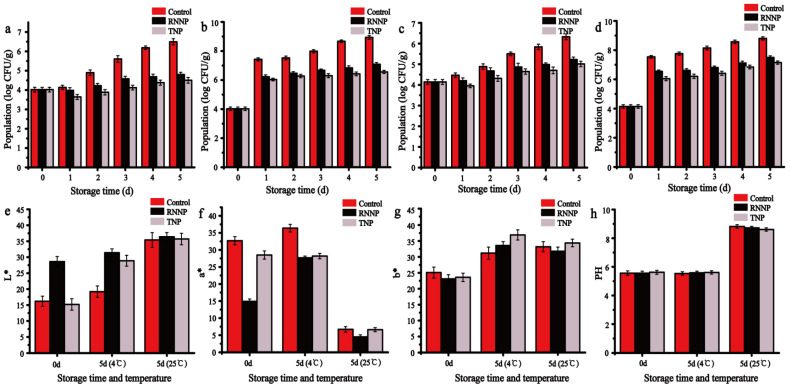
The application of nanoparticles in beef including antibacterial activity of RNNP and TNP against *S. aureus* at 4 °C (**a**) and 25 °C (**b**); The antibacterial activity of RNNP and TNP against *E. coli* O157:H7 at 4 °C (**c**) and 25 °C (**d**); color analysis of beef treated with RNNP and TNP at 4 °C and 25 °C (**e**–**g**); PH of beef treated with RNNP and TNP at 4 °C and 25 °C (**h**).

The long-term antibacterial activity of nanoparticles against *S. aureus* and *E. coli* O157:H7 on beef was presented in Figure 7a–d. The initial number of *S. aureus* was 4.024 log CFU/g. The beef was stored at 4 °C for five days. The amount of *S. aureus* in the control samples increased to 6.496 log CFU/g, the samples treated with RNNP increased to 4.802 log CFU/g, which was a 26.08% decrease compared to the control samples, and the samples treated with TNP increased to 4.505 log CFU/g, a 30.65% decrease (Figure 7a). When it was cultured at 25 °C for five days, compared with the control samples, the number of *S. aureus* in the samples treated with RNNP decreased 1.85 log CFU/g, and the samples treated with TNP decreased 2.374 log CFU/g (Figure 7b). When the nanoparticles were applied to beef with *E. coli* O157:H7, the number of *E. coli* O157:H7 was significantly increased in the control samples, with the initial population of 4.142 log CFU/g reached 6.325 log CFU/g at 4 °C and 8.803 log CFU/g at 25 °C. When the beef was stored at 4 °C for five days, the beef treated with TNP showed the lowest microbial population (5.013 log CFU/g), which was obviously lower than the samples treated with RNNP (5.225 log CFU/g) (Figure 7c). When it was cultured at 25 °C for five days, compared with the control samples, the number of *E. coli* O157:H7 in the samples treated with RNNP decreased by 14.78%, and the samples treated with TNP decreased by 18.51% (Figure 7d). These results indicated that the polysaccharide modification improved the antibacterial effect of RNNP, and the results were consistent with the inhibition zone and the time-kill curve.

The effect of nanoparticles on the color of beef was studied in Figure 7e–g. The L* value of beef in the samples treated with RNNP increased, the a* value decreased, and the b* value had no difference on day 0. When the beef was treated with TNP, TNP reduced the a* value of beef and had no effect on the L* value and b* value. When the beef was stored at 4 °C for five days, compared with the control group, the L* value of the group treated with nanoparticles increased, but there were no significant differences between the a* value and b* value. When it was stored at 25 °C for five days, there were no significant difference in the L* value, a* value, or the b* value of the group treated with nanoparticles compared with the control group. Therefore, the nanoparticles will not affect the color of beef.

The effect of nanoparticles on the PH of beef was shown in Figure 7h. It can be seen that the beef treated with nanoparticles did not change its pH value on the day zero. When the beef was stored at 4 °C for five days, the pH value of the control group and the group treated with nanoparticles did not change much compared with day zero. The result may be the result of the increase in pH after the beef has been acidified. When it was stored at 25 °C for 5 days, the pH value increased to 8.82 ± 0.02, 8.72 ± 0.01, 8.61 ± 0.02, respectively, and the increase rate of pH in the samples treated with nanoparticles was lower than that of the control group. These results showed that nanoparticles can inhibit the growth of microorganisms, and the antibacterial effect of TNP was better than that of RNNP.

The effect of nanoparticles on the texture of beef after five days of storage at 4 and 25 °C was presented in Table 2. The adhesiveness, resilience and cohesion of beef treated with RNNP increased significantly, and the resilience and cohesion of beef treated with TNP decreased significantly on day zero. When the beef was stored at 4 or 25 °C for 5 days, the springiness and gumminess of beef had no significant change. Other values of the group treated with nanoparticles were better than the control group. These results showed that nanoparticles could delay the change in the texture of the beef.

## 4. Conclusions

In this paper, we aim to prepare TNP with high encapsulation efficiency and high stability to make up for the instability of protein nanoparticles. This is a new type of TNP containing dual active ingredients, with better antibacterial and antioxidant activities. TNP were prepared by hydrophobic interaction and electrostatic interaction using REO as antibacterial materials, nisin as antibacterial materials and peptide-based materials of nanoparticles, and LBP as modified materials. TNP had good particle size (211.5 nm), better dispersion (PDI = 0.241), and a uniform earth shape. The encapsulation efficiency of TNP reached 32%. UV and fluorescence spectra showed that the increase in the encapsulation efficiency of TNP might be caused by the interaction between LBP and nisin, which exposed more water dispersing bonds. After the addition of LBP, the physical stability, thermal stability and storage stability of TNP were significantly improved. The application of TNP on beef presented a favorable preservation effect without affecting its color and texture. Therefore, TNP are suitable carriers of active substances and have a wide range of application prospects.

## Figures and Tables

**Table 1:** The Particle size, PDI and encapsulation efficiency of nanoparticles.

Nanoparticles	Nisin:LBP (V/V)	Particle Size (nm)	PDI	Encapsulation Efficiency (%)
Nanoparticles-I	1:0	167.8 ± 4.8	0.242 ± 0.008	53.7 ± 0.6
Nanoparticles-II	3:1	386.5 ± 2.3 ^a^	0.302 ± 0.022 ^a^	84.4 ± 0.3 ^c^
Nanoparticles-III	1:1	211.5 ± 8.1 ^d^	0.241 ± 0.002 ^c^	86.6 ± 0.2 ^a^
Nanoparticles-VI	1:3	266.7 ± 7.7 ^b^	0.269 ± 0.005 ^b^	86.4 ± 0.1 ^a^
Nanoparticles-V	1:5	256.3 ± 8.4 ^c^	0.227 ± 0.015 ^c^	85.6 ± 0.3 ^b^

Values are expressed as mean ± SD. ^a–d^ Different superscript within the same column represent significant differences (*p* ˂ 0.05).

**Table 2:** Effect of nanoparticles on the quality of beef after five days of storage at 4 °C and 25 °C.

Treatment	Texture Parameters							
		Hardness (g)	Adhesiveness (g.sec)	Resilience (%)	Cohesion	Springiness (%)	Gumminess	Chewiness
0 d	Control	25.916 ± 0.482 ^a^	−47.244 ± 1.572^c^	47.372 ± 2.758 ^b^	0.801 ± 0.003 ^b^	51.237 ± 2.573 ^a^	8.235 ± 0.628 ^a^	3.514 ± 0.089 ^b^
	RNNP	25.985 ± 0.893 ^a^	−19.499 ± 0.724 ^a^	51.061 ± 5.641 ^a^	0.853 ± 0.004 ^a^	53.423 ± 3.091 ^a^	8.703 ± 0.773 ^a^	3.825 ± 0.068 ^a^
	TNP	26.328 ± 0.737 ^a^	−26.424 ± 0.857 ^b^	40.243 ± 2.986 ^c^	0.763 ± 0.002 ^c^	47.279 ± 2.942 ^a^	8.255 ± 0.395 ^a^	3.301 ± 0.046 ^c^
5 d (4 °C)	Control	18.855 ± 0.961 ^b^	−41.829 ± 1.147 ^c^	38.297 ± 2.675 ^a^	0.597 ± 0.002 ^c^	39.582 ± 1.862 ^a^	6.425 ± 0.413 ^a^	2.125 ± 0.031 ^c^
	RNNP	20.275 ± 0.725 ^b^	−13.351 ± 1.426 ^a^	45.847 ± 4.842 ^a^	0.752 ± 0.006 ^a^	43.51 ± 3.427 ^a^	7.214 ± 0.386 ^a^	2.532 ± 0.073 ^a^
	TNP	23.016 ± 0.515 ^a^	−25.934 ± 0.984 ^b^	38.961 ± 2.967 ^a^	0.665 ± 0.005 ^b^	40.21 ± 2.074 ^a^	7.013 ± 0.514 ^a^	2.289 ± 0.062 ^b^
5 d (25 °C)	Control	11.616 ± 0.472 ^c^	−34.455 ± 0.963 ^c^	30.566 ± 1.653^b^	0.468 ± 0.002 ^c^	32.221 ± 1.143 ^a^	5.438 ± 0.327 ^a^	1.752 ± 0.057 ^c^
	RNNP	14.734 ± 0.357 ^b^	−10.345 ± 1.273 ^a^	40.968 ± 3.547 ^a^	0.638 ± 0.004 ^a^	37.061 ± 1.984 ^a^	6.217 ± 0.551 ^a^	2.316 ± 0.071 ^a^
	TNP	19.564 ± 0.416 ^a^	−21.362 ± 1.527 ^b^	36.805 ± 1.604 ^a^	0.615 ± 0.008 ^b^	35.415 ± 2.573 ^a^	5.941 ± 0.426 ^a^	2.046 ± 0.054 ^b^

Values are expressed as mean ± SD. ^a–c^ Different superscript within the same column represent significant differences (*p* ˂ 0.05).

## Data Availability

Not applicable.
